# Health literacy on tuberculosis prevention and control among people living with HIV: a cross-sectional study

**DOI:** 10.3389/fpubh.2026.1811550

**Published:** 2026-04-16

**Authors:** Mingkuan Fan, Xia Yuan, Chuangui Nie, Xian Wang, Wen Cheng, Qiangxiang Zhang

**Affiliations:** 1 Medical College, Xiangyang Polytechnic University, Xiangyang, Hubei, China; 2 Xiangyang Tuberculosis Prevention and Treatment Hospital, Xiangyang, Hubei, China

**Keywords:** acquired immune deficiency syndrome, cross-sectional studies, health behaviors, health education, tuberculosis

## Abstract

**Background:**

Tuberculosis (TB) remains a severe public health threat in China. People living with HIV (PLWH) face markedly higher risks of TB and TB-related mortality. Evaluating TB knowledge in this vulnerable group is critical for targeted health education, yet relevant data remain scarce. This study assessed TB awareness among PLWH to inform tailored TB health-education interventions.

**Methods:**

A cross-sectional survey was conducted using random proportional sampling from October to December 2024 in Xiangyang City, Hubei Province. A total of 225 PLWH completed face-to-face questionnaires covering sociodemographic characteristics, awareness of the five core TB information items, willingness to learn, current knowledge sources, and preferred information channels.

**Results:**

The overall awareness rate of core TB information among PLWH was 57.1%. Awareness varied significantly across specific items, ranging from 86.7% for “TB is an infectious disease” to only 28.0% for “TB is curable.” Lower awareness was significantly associated with rural residence (53.5% vs. urban 62.8%, *p* = 0.002), lower educational attainment (45.6% for junior high school or below vs. 69.4% for high school or above, *p* < 0.001), and being married or divorced/widowed compared to being unmarried (*p* < 0.001). Nearly all participants (98.7%) expressed willingness to learn about TB. Television was the most common current knowledge source (57.3%), whereas online media (e.g., WeChat, Douyin) was the most preferred channel (60.0%), followed by health lectures (48.0%).

**Conclusion:**

TB knowledge among PLWH was insufficient, with significant gaps in transmission, prevention, and curability. The vast majority demonstrated willingness to acquire TB knowledge and future efforts are needed to prioritize the underserved subgroups (e.g., rural and less-educated individuals). Digital platforms can be effectively combined with TB education integrated into routine HIV services. Messaging must focus on transmission, prevention, and curability through standard treatment adherence.

## 1 Introduction

Tuberculosis (TB) is a chronic infectious disease caused by *Mycobacterium tuberculosis* and continues to represent one of the most pressing global public health challenges (1, 2). According to the 2024 World Health Organization (WHO) report, an estimated 10.8 million new TB cases and 1.25 million TB-related deaths occurred worldwide, establishing TB as the leading cause of death from a single infectious agent (3). Approximately 62% of these new cases are concentrated in the WHO Southeast Asia and Western Pacific regions. As one of the 30 high-TB-burden countries, China accounts for 6.8% of the global incident TB cases, underscoring the substantial challenges facing national TB prevention and control efforts (3).

Public awareness of TB is a critical component of TB control strategies. A higher level of public awareness promotes policy responsiveness and mitigates TB-related discrimination and stigma (4). Crucially, it is a key determinant in facilitating early case detection, ensuring timely health-seeking behavior, and securing completion of standardized treatment (5, 6), all of which are essential for effectively interrupting community transmission. Since 2016, China has implemented a national TB prevention and control plan and a national TB action plan to achieve the goals of the WHO End TB Strategy. Both plans have established clear objectives for public TB awareness (7).

Compared with the general population, people living with HIV (PLWH) face a substantially higher risk—dozens of times greater—of developing TB following infection (8), and TB remains a leading cause of death in this group (3, 9). Acquiring essential TB knowledge, including transmission routes, key symptoms, and preventive measures, is therefore critical for enhancing prevention awareness, promoting protective behaviors, enabling early diagnosis, and reducing the TB-related disease burden among PLWH. However, relevant research on TB knowledge awareness among PLWH is limited both domestically and internationally, and localized data specifically from Central China remain particularly scarce, hindering the development of targeted and effective health-education interventions. To address this gap, this study aimed to investigate TB awareness and identify knowledge gaps among PLWH, offering a scientific foundation for developing tailored health education and promotion strategies.

## 2 Methods

### 2.1 Study design and settings

This cross-sectional study, based on face-to-face questionnaire surveys, was conducted from October to December 2024 in Xiangyang City, a prefecture-level city located in Hubei Province, central China. The study targeted PLWH aged 15 years or older and under routine follow-up in Xiangyang City, which comprised 9 county-level administrative divisions and had approximately 2,500 follow-up-eligible PLWH (10).

### 2.2 Survey methods and data collection

A random proportional sampling strategy was adopted. The sample size was calculated using Zstats v1.0,^1^ based on the 2022 national overall TB awareness rate of 84.2% among individuals aged ≥ 15 years (11), with a predefined margin of error and a significance level (*α*) of 0.05. The calculation yielded a minimum required sample size of 205 participants. Considering an expected 15% combined rate of loss to follow-up and non-response, a target sample size of at least 242 participants was set. Participants were then randomly selected in proportion to the distribution of all eligible PLWH under follow-up across 9 county-level administrative divisions.

Face-to-face interviews were administered by trained interviewers who completed a standardized training session prior to data collection. The training covered study objectives, item-by-item explanations of the questionnaire, and consistent probing techniques. A structured administration protocol with clear response options was followed to ensure uniformity across interviewers. Before each interview, the study purpose was explained thoroughly to participants, and verbal consent was obtained. Upon completion, a second staff member reviewed each questionnaire for consistency and completeness; any discrepancies were promptly communicated to the interviewer for re-verification with the participant as needed.

### 2.3 Questionnaire design

The questionnaire was adapted from a previously validated awareness assessment instrument used in the national “13th Five-Year” Tuberculosis Prevention and Control Program (12), and was finalized through a systematic literature review and expert consultation with TB control specialists. It consisted of three sections: (1) sociodemographic characteristics of participants (e.g., age; sex; education level; and occupation); (2) awareness of five core TB information items released by the Chinese health administration (13), which were: (i) TB is a chronic infectious disease; (ii) TB is primarily transmitted through the respiratory tract; (iii) a persistent cough and expectoration lasting more than 2 weeks are suspected TB symptoms; (iv) avoiding spitting, covering the mouth and nose when coughing or sneezing, and wearing masks can reduce TB transmission; and (v) TB is a curable disease in the vast majority of cases; (3) respondents’ willingness to learn about TB-related knowledge, their current sources of TB information, and their preferred channels for receiving such health education.

To assess the face validity and comprehensibility of the adapted questionnaire, a pilot survey was conducted among 8 PLWH recruited from urban Xiangyang, who were not included in the formal study sample. Participants were asked to complete the draft questionnaire and provide feedback on item clarity and wording appropriateness. The final version of the questionnaire was revised and optimized based on this pilot feedback (Supplementary file S1).

### 2.4 Ethical approval

Ethics approval was obtained from the Ethics Committee of Xiangyang Tuberculosis Prevention and Treatment Hospital. Verbal informed consent was obtained from all participants. The study posed no more than minimal risk to participants and did not collect any personally identifiable information, and all data were fully anonymized prior to data entry and statistical analysis to protect participant privacy.

### 2.5 Statistical analysis

All data were double-entered and cross-checked in an EpiData 3.1 database to minimize input errors, and statistical analyses were performed using R software (version 4.4.1). Continuous variables were presented as mean ± standard deviation, whereas categorical variables were summarized as frequency (*n*) and percentage (%). Descriptive statistics were computed for participants’ sociodemographic characteristics, awareness rates of core TB information, and TB information-seeking behaviors, including willingness to learn, current information sources, and preferred access channels.

Awareness rates were defined using standardized criteria. The single-item awareness rate was calculated as the percentage of respondents who answered the specific core TB item correctly. The overall awareness rate was derived from the total number of correct responses across all five core information items divided by the total number of possible correct responses for all participants. Between-group comparisons were performed using the chi-square test, with a *p*-value < 0.05 considered statistically significant (14).

## 3 Results

### 3.1 Characteristics of participants

A total of 242 PLWH were enrolled in the study. After excluding 17 cases due to loss to follow-up and non-response, 225 participants were finally included, yielding a response rate of 93.0%. The mean age of the participants was 49.9 ± 11.1 years; 114 (50.7%) were male and 111 (49.3%) were female. The majority of participants (61.3%) resided in rural areas, and 97.3% were of Han ethnicity. In terms of educational attainment, 52.0% had a junior high school education or below, while 108 participants (48.0%) had attained a high school education or higher. Agricultural workers constituted 48.0% of the study population, and two-thirds of the participants were married. Regarding monthly personal income, 117 participants (52.0%) reported an income of ≤ 2,999 Chinese Yuan Renminbi (RMB, the official currency of China) (Table 1).

**Table 1:** Sociodemographic characteristics of the participants (*N* = 225).

Characteristics	*n*	%
Age, year		
15–59	186	82.7
≥60	39	17.3
Sex		
Male	114	50.7
Female	111	49.3
Residence		
Urban	87	38.7
Rural	138	61.3
Ethnicity		
Han Chinese	219	97.3
Other ethnicities	6	2.7
Educational level		
Junior high school or below	117	52.0
High school or above	108	48.0
Occupation		
Agricultural worker	108	48.0
Unemployed/job-seeking	45	20.0
Other*	72	32.0
Marital status		
Unmarried	21	9.3
Married	150	66.7
Divorced/widowed	54	24.0
Monthly income, Chinese Yuan (RMB)		
≤2,999	117	52.0
3,000–5,999	90	40.0
≥6,000	18	8.0

RMB, Renminbi-the official currency of China; *Including commercial service workers, technical personnel, industrial workers and students.

### 3.2 Awareness of core TB information

The overall awareness rate of core TB information among all surveyed PLWH was 57.1%. Specifically, 86.7% of the respondents recognized that TB is an infectious disease; however, only 57.3% were aware of the primary transmission route of TB, nearly a quarter could not identify suspected TB symptoms, only 37.3% knew effective preventive measures against TB transmission, and merely 28.0% understood that TB is curable (Table 2).

**Table 2:** Awareness of core information on TB among people living with HIV (*N* = 225).

Core TB information item	Correct responses, *n*	Awareness rate, %
(1) TB is a chronic infectious disease	195	86.7
(2) TB is primarily transmitted through the respiratory tract	129	57.3
(3) A persistent cough and expectoration lasting more than two weeks are presumptive symptoms of TB	171	76.0
(4) Avoiding spitting, covering the mouth and nose when coughing or sneezing, and wearing masks can reduce TB transmission	84	37.3
(5) TB is a curable disease in the vast majority of cases	63	28.0
Overall	642	57.1

No statistically significant differences in overall TB awareness rates were observed according to sex, ethnicity, or personal monthly income (all *p* > 0.05). Participants residing in urban areas had a higher awareness rate than those residing in rural areas (62.8% vs. 53.5%, *p* = 0.002). Individuals with a high school education or higher had a markedly higher awareness rate than those with junior high school education or below (69.4% vs. 45.6%, *p* < 0.001). Differences in awareness rates were observed across occupation categories (*p* < 0.001), with the highest awareness rate in the “other occupations” group (71.7%), followed by agricultural workers (50.6%) and the unemployed/job-seeking group (49.3%). Similar differences were observed across marital status categories (*p* < 0.001), with the highest rate among unmarried participants (82.9%), compared to married (53.2%) and divorced or widowed (57.8%) participants (Table 3).

**Table 3:** Disparities in tuberculosis awareness by sociodemographic characteristics among people living with HIV (*N* = 225).

Characteristics	Number	Correct responses, *n*	Overall awareness rate, %	*χ* ^2^	*p*-value
Age, year				0.999	0.318
15–59	186	537	57.7		
≥60	39	105	53.9		
Sex				2.732	0.098
Male	114	339	59.5		
Female	111	303	54.6		
Residence				9.379	0.002
Urban	87	273	62.8		
Rural	138	369	53.5		
Ethnicity				0.108	0.742
Han Chinese	219	624	57.0		
Other ethnicities	6	18	60.0		
Educational level				64.938	<0.001
Junior high school or below	117	267	45.6		
High school or above	108	375	69.4		
Occupation				46.157	<0.001
Agricultural worker	108	273	50.6		
Unemployed/job-seeking	45	111	49.3		
Other*	72	258	71.7		
Marital status				33.138	<0.001
Unmarried	21	87	82.9		
Married	150	399	53.2		
Divorced/widowed	54	156	57.8		
Monthly income, Chinese Yuan (RMB)				2.881	0.237
≤2,999	117	330	56.4		
3,000–5,999	90	267	59.3		
≥6,000	18	45	50.0		

RMB, Renminbi-the official currency of China; *Including commercial service workers, technical personnel, industrial workers and students.

### 3.3 Willingness to acquire TB information

Among the 225 PLWH included in the analysis, 147 (65.3%) expressed a strong willingness to acquire TB-related information, 75 (33.3%) expressed a moderate willingness, and 3 (1.3%) expressed no willingness at all.

### 3.4 Sources of TB information acquisition

**Figure 1:**
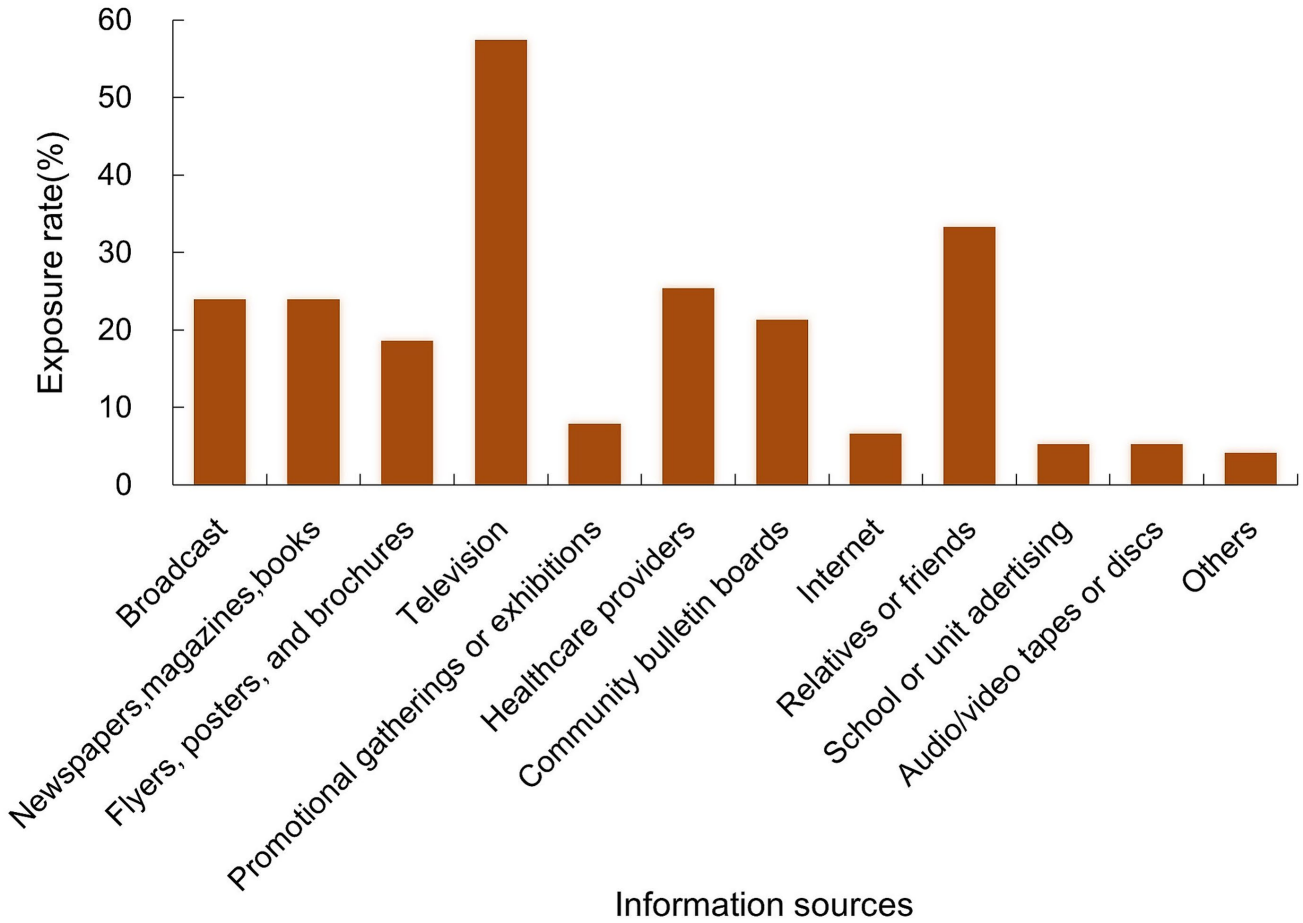
Information sources on TB among people living with HIV. Exposure rate: The proportion of participants who reported being exposed to TB information.

Participants accessed TB information through multiple sources (Figure 1). Television was the most frequently reported source (57.3%), followed by relatives or friends (33.3%) and healthcare providers (25.4%). Broadcast media and printed materials including newspapers, magazines or books were each reported by 24.0% of participants. Community bulletin boards accounted for 21.3%, while flyers, posters or brochures accounted for 18.7%. Promotional gatherings or exhibitions were indicated by 8.0% of participants, the Internet by 6.7%, and school or workplace advertising and audio/video tapes or discs by 5.3% each. Other sources were noted by 4.0% of participants.

### 3.5 Preferred channels for TB information acquisition

**Figure 2:**
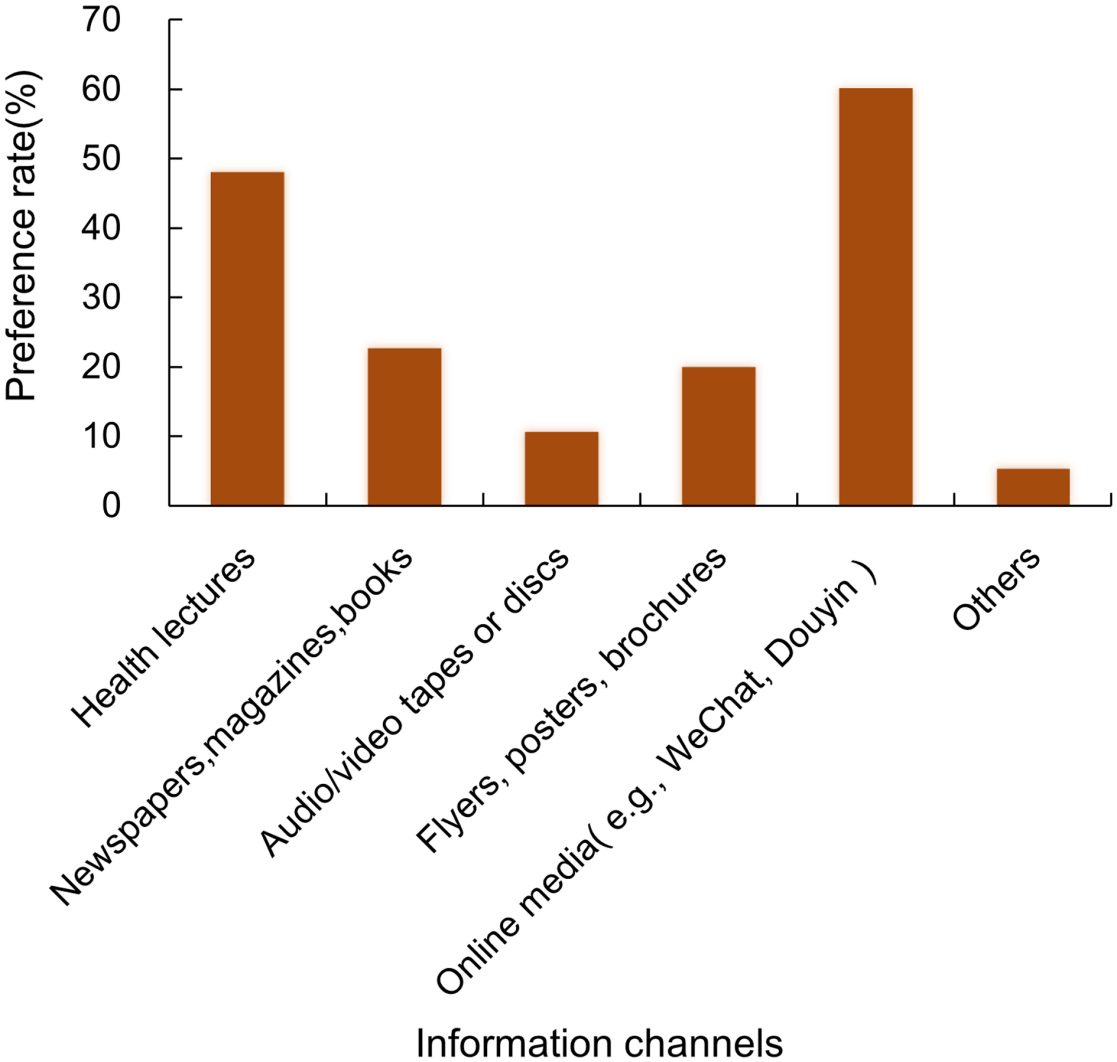
Preferred information channels for TB acquisition among people living with HIV. Preference rate: The proportion of participants who preferred a given TB information channel among all participants surveyed.

Online media platforms (e.g., WeChat, Douyin) were the most preferred channel (60.0%), followed by health lectures (48.0%). Newspapers, magazines, or books were preferred by 22.7% of participants; flyers, posters, or brochures by 20.0%; and audio/video tapes or discs by 10.7%. A small proportion of participants (5.4%) indicated a preference for other unspecified channels (Figure 2).

## 4 Discussion

Our findings demonstrated a notably low overall core TB information awareness rate (57.1%) among enrolled PLWH, which fell substantially short of the 85% national target set in China’s 13th Five-Year Plan for TB Control and the Tuberculosis Prevention and Control Action Plan (2019–2022) (15, 16). This rate was also lower than the 2022 national general public TB awareness rate of 84.2% and the 2020 general public awareness rate of 81.87% in Hubei Province (11, 17). Similar findings have been reported in studies conducted in India, Peru, and Thailand, all of which concluded that PLWH generally lack adequate knowledge regarding TB (18–20). One plausible explanation for the low awareness level in this study is that PLWH may experience varying degrees of social isolation due to disease-related stigma. Such stigma-related isolation can further limit their access to TB-related health information (21). Nevertheless, this hypothesis requires further targeted investigation for validation.

In the current survey, awareness of the statement “TB is an infectious disease” was the highest among the five core information items, reaching 86.7%, a result consistent with previous domestic studies focusing on the general population (11, 17). Based on this finding, it is reasonable to infer that the proportion of PLWH who have heard of TB exceeds 86.7%. In contrast, a 2008 survey among PLWH in India reported that only 69% of participants had heard of TB (18). The higher basic awareness observed in this study may be attributed to the continuous and extensive TB health education promotion implemented across China in recent years. Furthermore, differences in socioeconomic context, public health investment and health education coverage between the two countries (22), as well as discrepancies in study periods, may also contribute to the divergent findings.

Moreover, a marked disparity was identified between the TB knowledge structure of PLWH in the current study and that of the general population. While awareness of suspected TB symptoms (76.0%) was relatively high—likely due to routine TB screening integrated into HIV care services—awareness rates of transmission routes, preventive measures and disease curability (57.3, 37.3 and 28.0%, respectively) were substantially lower than national and provincial benchmarks in China (11, 17). These rates also fell below those reported in general population studies conducted in Uganda and Ethiopia (23, 24). This pattern indicates that although most PLWH hold a basic awareness that TB is an infectious disease, their understanding remains superficial. Critical knowledge regarding disease transmission, effective prevention strategies and treatment curability is notably lacking.

Such knowledge gaps carry important clinical and public health implications. They also suggest that routine TB screening for PLWH may not be fully implemented in practice, or that screening services are not accompanied by sufficient targeted TB health education (25). The widespread deficits in understanding TB curability and transmission routes likely reflect a phenomenon of knowledge fragmentation: individuals may recognize TB and its common symptoms, but lack comprehensive comprehension of its epidemiological characteristics and treatment outcomes. These gaps can lead to the adoption of ineffective or incorrect preventive behaviors, or excessive fear of TB infection (26). These findings highlight an urgent need to develop tailored TB health-education interventions for PLWH, with a specific focus on core essential knowledge: transmission routes, preventive measures, and the curability of TB through standardized and consistent treatment adherence.

The present analysis found that PLWH residing in urban areas had significantly higher TB awareness rates than their rural counterparts, a result consistent with both domestic and international relevant studies (26–29). This disparity may be attributed to inherent characteristics of rural settings, including dispersed population distribution that hinders efficient health information dissemination, and potentially limited accessibility to healthcare and health-education services (30, 31). From an intervention perspective, these results indicate that geographically targeted and region-adapted TB educational initiatives are warranted.

A well-documented positive correlation exists between educational attainment and TB health knowledge (26). Our findings support this existing evidence. Such educational disparities in TB knowledge may be explained by the fact that higher educational levels enhance individuals’ ability to comprehend, absorb and retain health-related information (32–34). Hence, future TB prevention education needs adopt differentiated communication strategies adapted to participants’ educational backgrounds.

Analysis of occupational differences revealed significant disparities in TB awareness levels. The “other occupations” group (including commercial service workers, technical personnel, industrial workers and students) showed the highest TB awareness rate, presumably attributed to their higher educational attainment and better health literacy relative to agricultural workers and unemployed individuals. This finding supports previous domestic studies (35, 36). Given the lower awareness among agricultural workers and unemployed subgroups, targeted TB health education should prioritize these vulnerable populations in future practice.

TB awareness was significantly higher among unmarried PLWH than among married, divorced, or widowed participants. Similar findings have been reported in studies conducted in Bangladesh and a district in central China (28, 37). This phenomenon may be partially attributable to the younger age and higher educational attainment of unmarried individuals, factors that enable better access to health information. However, an opposite conclusion was reported by Kaaffah et al. (38) in a multicenter survey conducted in Indonesia. Therefore, further studies are warranted to clarify the specific mechanisms by which marital status, combined with social and structural factors, influences TB information uptake among PLWH.

Nearly all participants reported moderate to high willingness to obtain TB-related knowledge. This proportion was slightly higher than that among medical college freshmen in a domestic study (39), suggesting a favorable basis for targeted TB education. Accordingly, future interventions should capitalize on this high motivation via accessible, engaging channels to convert willingness into effective knowledge acquisition. Television was the leading current information source (57.3%), likely owing to its wide coverage and themed TB campaigns around World Tuberculosis Day. While effective for population-level awareness raising, television is limited by fixed schedules and one-way communication, making it more suitable as a supplementary channel for basic TB publicity.

Regarding preferred information channels, nearly 60% of participants ranked online platforms such as WeChat and Douyin as their top choice, reflecting rising access to digital tools and a gradual shift away from traditional media (television, newspapers, radio, and audiovisual resources) (40, 41). Beyond wide population coverage, online media feature scalability and cost-effectiveness, enable real-time content updates, and reach geographically dispersed groups, including rural residents with limited access to conventional health education (42). Accordingly, digital platforms should be prioritized as core channels for TB health education among PLWH and fully integrated into routine HIV care services. Perceived as systematic and authoritative, health lectures delivered by healthcare professionals ranked second in preference (48.0%), corresponding with existing literature (43).

Notably, the 2024—2030 National Tuberculosis Prevention and Control Plan prioritizes PLWH and sets a national TB awareness target of ≥ 85% (44). Combined with our findings, targeted strategies—including integrating standardized TB education into routine HIV care, adopting popular digital platforms, delivering tailored outreach for vulnerable subgroups, and conducting regular TB literacy monitoring—are urgently needed to strengthen targeted TB prevention among this high-risk population.

### 4.1 Strengths and limitations

This study provides rare evidence from a central Chinese city for TB-HIV integrated control, identifying a critical mismatch between actual and preferred TB information channels and informing targeted policy and field implementation. Despite these strengths, several limitations should be acknowledged. First, the sample was confined to a single prefecture-level city, limiting generalizability due to regional sociocultural heterogeneity. Second, the questionnaire was adapted from general-population instruments rather than tailored for PLWH, with no formal psychometric validation or inter-rater reliability assessment. This is critical, as PLWH often present with atypical TB symptoms; this mismatch may have introduced substantial measurement bias in awareness rates. Third, self-reported data may be subject to social desirability bias given the stigma associated with TB-HIV co-infection. To address these limitations, future research should include multi-site sampling, employ validated PLWH-tailored instruments with formal psychometric evaluation, and consider objective or qualitative methods.

## 5 Conclusion

TB health literacy among PLWH remains suboptimal, with substantial knowledge deficits regarding TB transmission, prevention, and curability. The majority of participants expressed willingness to acquire TB knowledge, indicating the feasibility of targeted health promotion interventions. Future tailored strategies should prioritize PLWH who are rural residents, less educated, agricultural workers, unemployed, or married/divorced/widowed. Digital platforms are recommended as primary educational channels, and TB education should be integrated into routine HIV care services, with focused content on TB transmission, effective prevention, and curability under standardized treatment.

## Data Availability

The original contributions presented in the study are included in the article/Supplementary material, further inquiries can be directed to the corresponding author.

## Footnotes

1
http://www.zstats.net
