# Supplementary material for: Health literacy on tuberculosis prevention and control among people living with HIV: a cross-sectional study
Source: Front Public Health. 2026 Apr 16;14:1811550. doi: 10.3389/fpubh.2026.1811550 (PMC13128581; doi:10.3389/fpubh.2026.1811550)
Supplement: Supplementary file 1 [file Data_Sheet_1.pdf]

# Tuberculosis Prevention and Control Knowledge Awareness Questionnaire

**Dear Respondent,**

We are staff from Xiangyang Tuberculosis Prevention and Control Hospital. This survey is conducted to understand your awareness of core knowledge regarding tuberculosis prevention and control. Your answers are of great significance for improving TB prevention and control work. We will strictly keep your personal information confidential. Please answer the questions truthfully based on your actual situation. We sincerely appreciate your support and cooperation!

**Ethical and Data Protection Information**

**Ethical Approval**: This study has been approved by the Ethics Committee Review Board of the Xiangyang Tuberculosis Prevention and Treatment Hospital. The studies were conducted in accordance with the local legislation and institutional requirements.

**Informed Consent**: Verbal informed consent for participation was required from the participants. Your participation in this survey is completely voluntary. By agreeing to participate and completing this questionnaire, you are providing verbal informed consent. You may refuse to answer any question and may withdraw from the survey at any time without any consequences.

**Data Protection**: All information you provide will be kept strictly confidential. Data will be anonymized and used solely for research purposes. Only authorized research staff will have access to the data. Your personal information will not be shared with any third party.

**Questionnaire Code**: □□□□□□

**Survey Location**: ________ County (District)

**Survey Date**: _____ Year _____ Month _____ Day

**Investigator (Signature)**: _____________

## Part 1: General Information

1. Your age: ______ years old

2. Your gender:

   1) Male

   2) Female

3. Your ethnicity:

   1) Han Chinese

   2) Ethnic minority

4. Your education level:

   1) Primary school and below

   2) Junior high school or equivalent

   3) Senior high school (technical school, secondary specialized school)

   4) College graduate

   5) Bachelor's degree and above

5. Your place of residence:

   1) Urban area

   2) Rural area

6. Your current marital status:

   1) Unmarried

   2) Married

   3) Divorced

   4) Widowed

7. Your occupation:

   1) Staff of government organs and enterprises/institutions

   2) Worker

   3) Farmer, herdsman, fisherman

4) Student

5) Commercial service personnel

6) Unemployed

7) Retiree

8) Other

---

## Part 2: Awareness of Core Tuberculosis Information

1. Is tuberculosis an infectious disease?

1) Don't know

2) No

3) Yes

2. Tuberculosis is mainly transmitted through which of the following ways?

1) Don't know

2) Coughing or sneezing at others

3) Shaking hands, hugging

4) Sharing tableware

5) Blood transfusion

3. Which of the following symptoms should most suggest the possibility of having tuberculosis?

1) Don't know

2) Cough, expectoration or blood in sputum for more than 2 weeks

3) Abdominal pain, diarrhea

4) Headache, dizziness

4. Which of the following can effectively prevent tuberculosis?

1) Don't know

2) Not eating unclean food and not sharing tableware

3) Not spitting anywhere, covering mouth and nose when coughing or sneezing, wearing masks in crowded public places

4) Not sharing toothbrushes, razors, towels and other items with others

5) Reducing contact with animals and avoiding mosquito bites

5. If receiving standardized treatment, can tuberculosis be cured?

1) Don't know

2) The vast majority can be cured

3) Cannot be cured

---

## Part 3: Willingness, Sources and Preferred Channels of Tuberculosis Knowledge Acquisition

1. What is your willingness to learn about tuberculosis knowledge?

1) Unwilling

2) Willing

3) Very willing

2. Where did you get your current tuberculosis knowledge? (Multiple choices allowed)

1) Broadcast

2) Newspapers, magazines, books

3) Flyers, posters, and brochures

4) Television

5) Promotional gatherings or exhibitions

6) Healthcare providers

7) Community bulletin boards

8) Internet

9) Relatives or friends

10) School or unit advertising

11) Audio/video tapes or discs

12) Others

3. Where do you hope to obtain tuberculosis prevention and control knowledge? (Multiple choices allowed)

1) Broadcast

2) Flyers, posters, brochures

3) Television

4) Promotional gatherings or exhibitions

5) Health lectures

6) Newspapers, magazines, books

7) Audio/video tapes or discs

8) Online media (e.g., WeChat, Douyin)

9) Relatives or friends

10) Others

**Thank you for your participation!**
